# Giant Intrapericardial bronchogenic cyst associated with congestive heart failure and atrial fibrillation: a case report

**DOI:** 10.1186/s13019-021-01412-2

**Published:** 2021-03-19

**Authors:** Javier Maldonado Escalante, German Molina, Francisco Mauricio Rincón, Lina M. Acosta Buitrago, Carlos J. Perez Rivera

**Affiliations:** Department of Cardiovascular Surgery, Clínica Universitaria Colombia, Carrera 23 #66-46, Bogota, DC Colombia

**Keywords:** Atrial fibrillation, Heart failure, Intrapericardial mass, Bronchogenic cyst

## Abstract

**Background:**

Large intracardiac bronchogenic cysts are rare mediastinal masses. However, they must always be considered in the differential diagnosis of heart failure with abnormal chest X-ray.

**Case presentation:**

We present a 60-year-old female patient with de novo atrial fibrillation, heart failure and a very large intrapericardial mass. The patient underwent successful surgical resection, with pathological findings confirming a bronchogenic cyst.

**Conclusions:**

Large bronchogenic cysts located intrapericardially are very rare. However, they should be included in the differential diagnosis of patients presenting with atrial fibrillation and heart failure with abnormal radiologic studies.

## Background

A bronchogenic cyst is a remnant of the primitive gut tube occurring as a consequence of abnormal embryonic development in the tracheobronchial tree between the 5th and 16th gestational week [1–3].

It is a rare congenital defect, with an incidence of 1/50000 people [4], representing 14–22% of all congenital pulmonary defects [2] and 10–15% of all primary mediastinal masses [1]. The average diameter ranges from 2 to 4 cm^2^ and is classified based on location as parenchymal or mediastinal, the latter being 86% of the cases. Intrapericardial and intracardiac locations are not common in the literature [5]. We present a case of a patient who underwent successful resection of a giant intrapericardial bronchogenic cyst of 8x6cm, described in accordance to surgical case report (SCARE) criteria [6].

## Case presentation

**Fig. 1:**
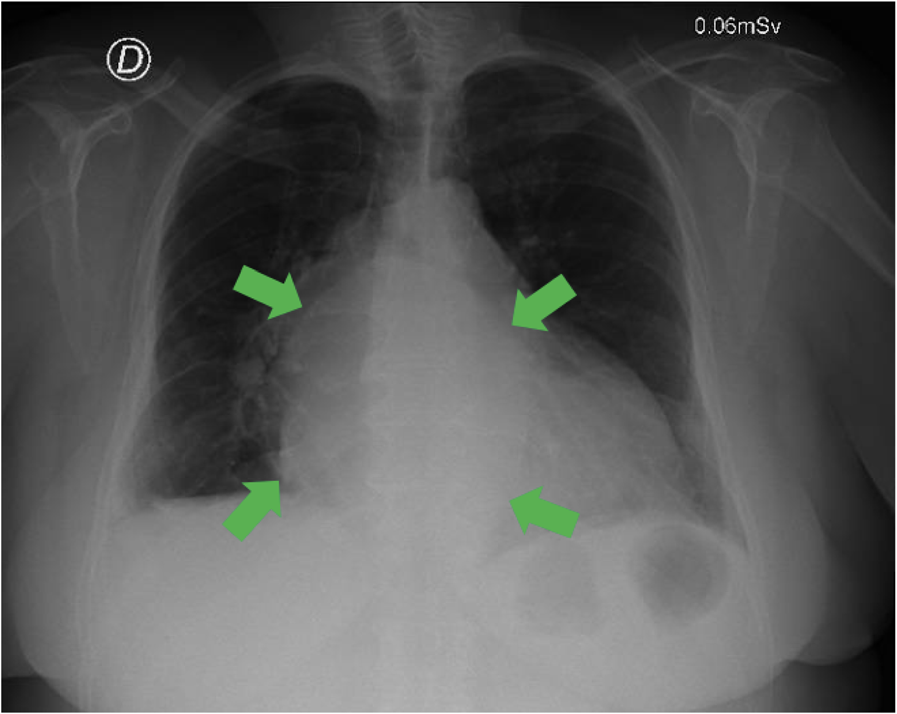
Chest X-Ray. Retrocardiac opacity indicated by green arrows, suggestive of a giant mass

A 66-year-old female patient, with no previous cardiovascular diseases, presented to the emergency room (ER) with fatigue and severe chest pain that worsened with exertion. The patient had the symptoms for a few months, but because of increased pain and dyspnea she decided to consult. Upon arrival, the electrocardiogram showed a de novo atrial fibrillation with a rapid ventricular response. The resulting chest X-ray revealed a poorly defined retrocardiac opacity suggestive of a giant mass [Fig. 1]. The transthoracic echocardiogram revealed a non-obstructive concentric hypertrophic left ventricle with moderate systolic disfunction – left ventricular ejection fraction (LVEF) of 35–40%, sclerosis of the mitral and aortic valve without hemodynamic compromise, mild tricuspid insufficiency, and moderate pericardial fluid. However, due to a very poor acoustic window, a transesophageal echocardiogram was suggested, with the coronary arteriography ruling out coronary disease. The transesophageal echocardiogram showed a LVEF of 51% with mild tricuspid insufficiency, moderate pulmonary hypertension, a moderate pericardial effusion, and multiple pericardial masses. She was started on diuretics (furosemide) and beta blockers that failed to control the heart rate. Consequently, administration of intravenous amiodarone was required.

**Fig. 2:**
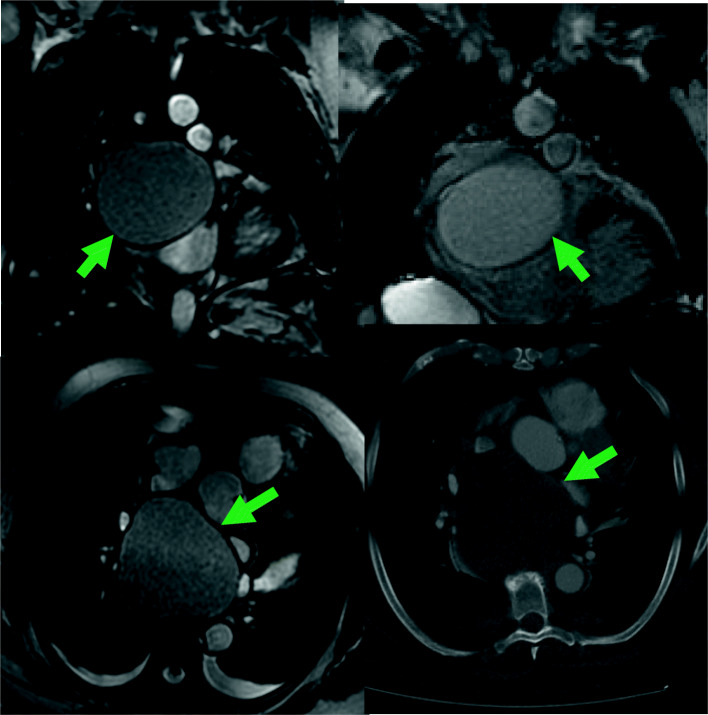
Cardiac MRI. Green arrows represent the dimensions and localization of the intrapericardial mass

The pericardial masses were further studied with a contrasted CT scan that showed a 10 cm compressive mass in the posteromedial mediastinum very suggestive of a pericardial cyst. A cardiac magnetic resonance image (MRI) defined a 96 × 88 × 77 mm mass in its transverse, anteroposterior and cephalo-caudal diameters, respectively. The mass was located in the superior pericardial ridge, in contact anteriorly with the aorta and pulmonary artery, laterally with the superior vena cava, posteriorly with the vertebral bodies, and inferiorly with the left atrium roof producing partial left atrial compression. The mass had a homogeneous high signal intensity in T2, without any changes in the opposite phase, and did not highlight with intravenous contrast [Fig. 2].

**Fig. 3:**
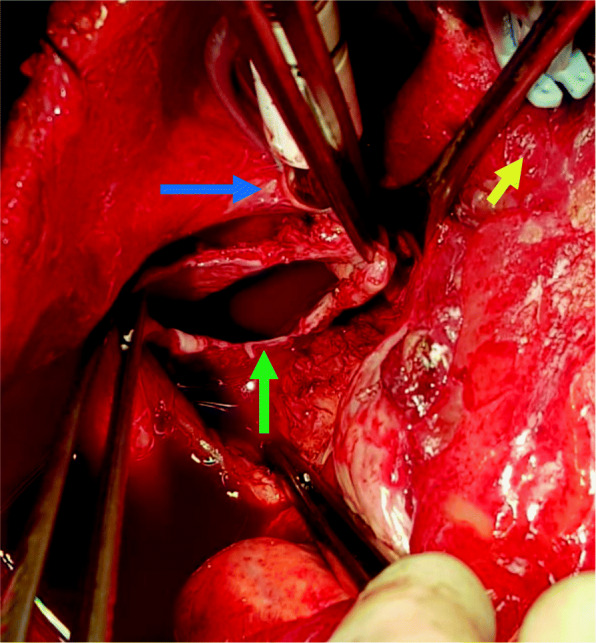
Intra-Operative Image. The open cyst (green arrow) showing the wall thickness, with the superior vena cava cannulation (blue arrow) and the aorta (yellow arrow)

After considering different surgical approaches (left thoracotomy and conventional sternotomy), the latter was chosen based on the CT scan and MRI results. Severe pericardial adhesions did not allow access to the mass, requiring conventional aortic and bi-caval extracorporeal circulation. After careful dissection, the 8 × 6 cm cyst was exposed. The cyst was firmly attached to the left atrial wall including the right superior and inferior pulmonary veins, superior vena cava and right pulmonary artery (Fig. 3). Surgery was completed, however. The full cyst resection could not be completed without extracorporeal circulation. Both the cyst pieces and a pericardial biopsy were sent to pathology.

After a 4-day intensive care unit (ICU) stay, the patient was transferred to the step down unit. A post-operative transthoracic echocardiogram showed a LVEF of 55% and no pericardial effusions. Clinically there was no atrial fibrillation nor any signs of congestive heart failure. She was discharged 3 days later. The macroscopic pathological report described an 8x6cm mass with a 0.3 cm wall thickness of fibroelastic consistency. The histological findings suggested a benign cystic lesion made up of fibromuscular wall and mature cartilage, outlined by ciliated columnar epithelium, consistent with a bronchogenic cyst. Patient follow-up at 1, 3, and 6-months later reported a Short From 36 (SF-36) quality of life scale of 95% (minimal disability).

On her first year out-patient follow-up she was asymptomatic on New York Heart Association class I. The check-up CT scan on this consult was completely normal.

## Discussion

Bronchogenic cysts are rare congenital malformations dependent on the anterior intestine [5]. This malformation can occur during the third and fourth embryonic development when the primitive gut tube divides dorsally into the embryonic esophagus, and ventrally into the embryonic pulmonary yolk and tracheo-bronchial tree [2]. When an intrapericardial bronchogenic cysts is formed, the heart and lungs share a common celomic cavity. Thus, the pulmonary yolk’s separation from the traqueo-bronquial tree and its inclusion into the pericardial cavity can occur simultaneously [5]. Based on the location, they can be classified as parenchymal or mediastinal (the latter being 86% of cases) [2]. Additionally, they may also be classified as precarinal (52%), paratracheal (19%), paraesophageal (14%), and retrocardiac (9%) [2]. Occasionally, they may occur in other locations such as subcutaneous or cutaneously either in the neck or diaphragm. However, the intrapericardial and intracardiac locations are extremely rare in the literature [5]. Their size often varies on average from 2 to 4 cm^2^ [Table 1].

**Table 1:** Reported Cases of Intrapericardial Bronchogenic Cysts in the Literature

Authors	Age (years)	Gender	Size (cm) L x W x H	Location	Treatment	Extracorporeal Circulation
Maldonado J et al.	66	Female	8 × 6 × 0.3	Pericardial	Resection	Yes
Qu X et al. [5]	1.6	Female	5.3 × 3.6 × 2.8	Pericardial	Resection	Not described
Li Z et al. [7]	17	Male	9 × 8.4	Left atrium	Resection	Yes
Olsen M et al. [8]	50	Female	3.4 × 3.3 × 4.1	Interatrial septum	Resection	Yes
Wang J et al. [9]	41	Female	2.5 × 1.5 × 2	Left ventricle	Resection	Yes
Borges AC et al. [10]	43	Female	4.4 × 3.4	Interatrial septum	Resection	Not described
Forcillo J et al. [11]	41	Female	1 × 1.7 × 1.2	Left ventricle	Resection	Yes
Nishida N et al. [12]	73	Male	0.5	Interventricular septum	Death	N/A

Often asymptomatic, 19% of cases are diagnosed as incidental findings on chest x-rays [2, 3]. Typical symptoms may include retrosternal chest pain, dyspnea, coughing, stridor, fatigue, weakness, anorexia and fever [2, 3, 5]. Clinical manifestations depend on the localization of the mass, its size and whether it exerts any compression on adjacent structures [5]. If the cyst is small enough, and avoid any adjacent structures, it is usually asymptomatic. When the cyst is large, it usually produces coughing, chest pain, difficulty breathing or even dysphagia. In adults, most cysts are tiny, reducing the chance of any ongoing symptoms [1]. However, in this case report we show a large cyst generating important clinical manifestations, including heart failure and a cardiac arrhythmia.

A CT-scan is used to identify location, size, shape, and its relationship with adjacent structures, unfortunately it is effective in only 10–40% of patients [13]. Other studies such as an MRI have higher diagnostic sensitivity given its high signal intensity in T2 images [14]. Additionally, MRI can be useful to determine the cyst’s origin, its relationship with other structures and can even determine the best surgical approach [13]. In this case report, the chest CT scan was not enough to provide an adequate characterization of the cyst’s walls and relation to adjacent cardiac structures, hence warranting a cardiac MRI.

The definitive diagnosis of a bronchogenic cyst is histopathological with the surgical resection [3]. Typical findings include ciliated columnar epithelial cells along the inner lining [14]. The internal wall can also contain cartilage and smooth muscle tissue, but these are not essential for the diagnosis. In this case, the cyst had ciliated columnar epithelial cells with fibromuscular tissue and mature cartilage of the inner wall, confirming the bronchogenic cyst diagnosis.

The best treatment is surgical resection in symptomatic patients in order to avoid late complications such as infection, rupture or malignity [2, 5]. Prognosis without treatment has a 100% mortality rate, which is reduced to 0–14% with surgical management [2]. In this case report, given the intrapericardial location, an extracorporeal circulation with bi-caval and aortic cannulation was required to ensure an adequate visualization and subsequent resection. In previous intracardiac and intrapericardial case reports, most required extracorporeal circulation for a successful resection.

## Conclusions

Although numerous bronchogenic cyst reports have been published, this is the first one with such a large sized intrapericardial and retrocardiac location. In patients presenting with heart failure, de-novo atrial fibrillation and abnormal chest X-ray, a mediastinal mass cannot be ruled out and should be considered within the differential diagnosis. The chest CT scan and cardiac MRI are excellent complementary images; however the definitive diagnosis is a histopathological examination of the cyst’s surgical extraction.

## Data Availability

The dataset supporting the conclusions of this article is included within the article, any other inquiry is available from the corresponding author on reasonable request.

## Abbreviations

SCARE: Surgical Case Report Guidelines
ER: Emergency Room
LVEF: Left ventricular ejection fraction
CT: Computed tomography
MRI: Magnetic resonance image
ICU: Intensive care unit
